# Supplementary material for: “It's disappointing and it's pretty frustrating, because it feels like it's something that will never go away.” A qualitative study exploring individuals’ beliefs and experiences of Achilles tendinopathy
Source: PLoS One. 2020 May 29;15(5):e0233459. doi: 10.1371/journal.pone.0233459 (PMC7259496; doi:10.1371/journal.pone.0233459)
Supplement: S1 Appendix — (DOCX) [file pone.0233459.s001.docx]

**S1 Appendix. Interview Question Route.**

**Introduction**

Thank you for agreeing to talk to me today. This interview is part of a project being conducted by Monash University. We want to talk to people who have had persistent Achilles tendon pain.

I would like to digitally record our conversation today if you agree. No identifying information will be placed in any verbal or written reports associated with our conversation. This information will help us to develop a better understanding about your knowledge, experience and beliefs about tendon pain. This information may provide insights into how physiotherapists could improve the management of chronic Achilles tendon pain.

| **Question** | **Prompt** |
| --- | --- |
| Please tell me all about your Achilles tendon pain. | When did it start?  Is it there all the time?  What makes it better?  What makes it worse? |
| What do you think initially caused your Achilles tendon pain? | Trauma (e.g. fall)  Specific movements or activity  Change in loading pattern  Ageing  How does it make you feel knowing (or not knowing) the cause of your Achilles pain? |
| What does your pain mean to you? | What do you think about when you are in pain?  What do you think the pain means?  Did you have scans?  If yes, were the scan findings explained?  What do you think the scans findings mean?  Most tendon pain heals and does not last for a long time, so why do you think your Achilles pain did not heal initially?  Why do you think some people get better and others don’t? |
| Please tell me how your Achilles pain affects your life | Explore impact on activities of daily living, work, leisure activities  Impact on relationships, social life, emotional impact (eg loss of self) due to reduced activity (e.g. walking, running)  What motivated you to seek treatment? |
| What treatment(s) have you tried for your Achilles pain? | Prompt exercise, advice about activity, education about your condition, hands on work, tapes, braces, electrotherapy machines, medications  Why did you choose these treatments?  What aspects were most helpful/not helpful? Why do you think this is the case?  What do you understand to be the treatments that are most effective  for treating Achilles tendon pain like yours? |
| What did the exercise part of your treatment involve? (if relevant) | Do you think exercise is helpful for Achilles pain? Any specific exercise? Why?  What is your understanding of why you were doing the exercise?  Did you do the exercises regularly? Why? |
| How do you see yourself in the next 1-2 years? | If better, why?  If worse/same, why? What will cause it to get worse/stay same?  Do you feel the tendon is still weak? (may prompts fear of rupture)  Do you feel in control of managing your Achilles pain from now on?  What do you do (exercise and other) to manage your Achilles pain now?  Do you believe your Achilles pain can be cured? Why? |
| Where have you gained information about Achilles tendon pain | Clinicians, online, books?  What was helpful? Why? Why not?  What information did you like the most? (prompt online, printed, written, images, audio, video)  Did these influence your choice of a particular treatment? |
| What have you learned from having AT pain? | What would you suggest to other people diagnose with AT?  Is there anything else that we have not discussed that you would like to share? |
