# Supplementary material for: “It's disappointing and it's pretty frustrating, because it feels like it's something that will never go away.” A qualitative study exploring individuals’ beliefs and experiences of Achilles tendinopathy
Source: PLoS One. 2020 May 29;15(5):e0233459. doi: 10.1371/journal.pone.0233459 (PMC7259496; doi:10.1371/journal.pone.0233459)
Supplement: S2 Appendix — (DOCX) [file pone.0233459.s002.docx]

**S2 Appendix. Audit Trail.**

| Institutional ethics board approval and oversight |
| --- |
| Ethical approval for the study was sought and granted by Monash University Human Ethics Committee (Ethics Number: 10006). |
| Literature review |
| A detailed literature review was undertaken as part of the research proposal. Given the distinct lack of qualitative research in the area of Achilles tendinopathy the literature review was focused on other peripheral musculoskeletal disorders such as the knee, hip or shoulder. An extensive review of the literature allowed authors to draw parallels and identify potential gaps to inform the research proposal. |
| Conceptual/theoretical framework |
| An inductive thematic analysis [1] was used to uncover participants experience of AT. Thematic analysis is a qualitative analytic method for identifying, analyzing and reporting patterns (themes) within data. It minimally organizes and describes the data set in (rich) detail while also providing flexibility in interpreting various aspects of the research topic. |
| Interview protocol and instrument development: |
| The questioning route and interview guide was drafted and agreed upon by all authors of the study. The questioning route followed was similar to that used by Mc Auliffe et al [2] in the only other previous qualitative study in AT. The questioning route explored: participants history of AT, perceived cause of AT, experience in managing AT, and perspective on future prognosis (For full interview guide see S1 Appendix). |
| Participant selection |
| A convenience sample method was performed in Melbourne, Australia via running clubs and from patients attending a large private practice specializing in the management of MSK disorders. Written consent was obtained prior to the interviews. Potential participants were athletic and non-athletic individuals, aged 18-75 years old with localized Achilles pain for >3 months duration of symptoms; gradual onset of Achilles tendon pain (subjective reporting); pain aggravated during or after weight-bearing activity; and evidence of Achilles thickening, hypoechoic regions, and/or Doppler signal on ultrasound imaging were eligible to participate in the study. Ultrasound imaging was performed by one experienced physiotherapist (PM) trained in ultrasound imaging and has imaged over 1000 Achilles tendons. Individuals with both mid portion and insertional tendinopathy were eligible to participate in the study.  “Potential participants were contacted via e-mail to invite participation in the study. Written  consent was obtained prior to interview. In instances where invitees did not respond or  refused to participate, we continued to invite additional suitable participates from our  sampling frame. Sampling continued until thematic saturation was achieved, with two cocoders agreeing that no new themes were emerging. |
| Data collection and storage |
| Semi-structured telephone interviews (15 interviews) were carried out by one of the authors (JG). The interviewer (JG) was a male physiotherapist (BPhysio) who was unknown to the participants and was guided by a flexible questioning route. Prior to conducting the interviews, the interviewer (JG) undertook several practice interviews with feedback provided by a member of the investigation team (SMA). Interviews lasted from approximately 30-60 minutes. Interviews were recorded using a digital voice recorder. |
| Raw data |
| Audio files using a digital voice recorder, filed notes and site documents (participant information sheet, consent form, baseline screening) were all stored in a secured location. |
| Partially processed data |
| Coded interview transcripts and key informant responses; write ups of observational comments, and researchers field notes. |
| Coding scheme |
| Interviews were transcribed verbatim. Data coding was performed using the six-step framework approach proposed by [1]: (Generating initial codes, searching for themes, reviewing themes, defining and naming themes, producing the report, producing the report). Initial code lists were then amalgamated, and a comprehensive code list was finalized in view of the codes most representative of the dataset informed by background reading related to the research question (SMA and JT). The finalized code list was then applied to all transcripts by two members of the group (SMA and JT). Coded data was categorized and through a process of repetitive interpretation, synthesizing and theorizing – themes were identified [3]. Transcripts were then re-read several times and the selected themes were finalized based on consensus discussion between three study authors (SMA, JT, and PM). |
| Trustworthiness techniques |
| In order to ensure trustworthiness of the data Guba’s Four Criteria for Trustworthiness (credibility, transferability, dependability, confirmability) were taken into consideration when undertaking the qualitative research. Some of the methods used to ensure trustworthiness of the research included:   - Member checking: Checks relating to the accuracy of the data were undertaken through member checking. Members were individually emailed their transcriptions and were provided with a set list of member check questions, which can be found in S2 Appendix. - Triangulation of data: In order to improve the validity of the data and research project triangulation of data was undertaken to capture different dimensions of the same phenomenon. All participants included in the current study also engaged in a intervention study which involved baseline assessment of psychological, general health and functional impairments relating to their AT, thus ensuring a variety of data collection methods to improve trustworthiness. - Debriefing sessions: JG, the individual involved in performing the interviews engaged in regular debriefing sessions with a member of the research team (SMA) to ensure credibility of the data collected. |
| Research manuscript |
| Following formation and generation of the interview themes using appropriate statistical methodologies a research manuscript was undertaken using a collaborative approach of the aforementioned study. Given the distinct lack of qualitative research in the area of tendinopathy the present study reported compared findings to qualitative research in the only other qualitative study in tendinopathy as well as literature pertaining to peripheral musculoskeletal disorders. Limitations and recommendations for future research based on the study findings were also discussed in the research report. |

**Cumulative Main Themes and Sub Themes**

1. Beliefs on AT
   1. Causal Beliefs
   2. Pain Beliefs/Meaning
   3. Fear Avoidance
   4. Treatment Beliefs
2. Experience with Management Process
3. Motivations to seek treatment
4. Barriers for treatment adherence
5. Experience with passive/active modalities
6. Treatment expectations
7. Self-management strategies
8. Impact of AT
   1. Impact on daily life
   2. Impact on physical activity
   3. Frustrations
   4. Self-blame
9. Prognosis
   1. Perception of future prognosis
   2. Perceptions surrounding tendon rupture
   3. Self-efficacy
   4. Advice to others
10. Desire for Education
    1. Preferred sources and format of information
    2. Education from HCP’s
    3. Preferred features of HCP’s

**Cumulative Main Themes, Sub Themes, and Quotes**

1. **Beliefs on AT**
   1. **Causal Beliefs**
      1. 10/15 participants believe overtraining/overuse is a primary cause.
      2. 11/15 participants believe a change in biomechanics is a primary cause.
      3. Participant 2: I wasn't sort of doing anything much injury prevention sort of work. So I guessing it's probably I kept pushing myself without giving myself much of a break or doing any strength work I guess to try and protect myself from that injury.”
      4. Participant 15: I just assume that I've become slightly unfit and that I always had tight muscles in my legs and it's kind of a consequence of decades of not really exercising.
      5. Participant 10 - So I feel that I probably over trained. Not so much leading up to the run, it was more I didn't recover and allow myself time to recover afterwards and I just pushed it a little bit too far.
      6. Participant 2 - And I think I just got ... bit off more than my body could chew. And I also wasn't doing a lot of strength work either, I was just running. So I wasn't sort of doing anything much injury prevention sort of work. So I guessing it's probably I kept pushing myself without giving myself much of a break or doing any strength work I guess to try and protect myself from that injury.
      7. Participant 10: Because I was over training and I didn't listen to my body.
      8. Participant 11: it's all about perhaps putting too much load on this tendon because normally it is probably designed to function okay.
      9. Participant 4: I'd only just started training a month before I went to Bali and then I think it was just too much too soon
   2. **Pain Beliefs/Meaning**
      1. 11/15 participants believe their persistent pain is caused by tendon weakness
      2. Participant 1 - It definitely is slowing muscle, so a muscle ... There’s been some damage to the muscle tissues and the surrounding area, that there’s a little swelling in that area. Because if you rest it and the swelling subsides, then the pain subsides.
      3. Participant 2: “So yes, I think these areas of my body are obviously weak so ... most calves are weak so my achilles have to pick up a bit of the load and other parts of my body are weak so I've got to strengthen those up”
      4. Participant 11 - Yeah, but there is some kind of inflammation, maybe the other muscles are too weak and do not cooperate with this tendon to help it maybe because the physio gave me some you know, stretchable, some rubber band that we try to make some small muscle stronger, something. It wasn't the main thing, but you know.
      5. Participant 8: Like the expectation's there so I guess the pain will be there. I've got to kind of break that that's what I'm thinking. I've got to break that sort of thinking.
      6. Participant 5: It's saying that it doesn't want to do what my mind is telling my body!
      7. Participant 5: In my mind is not to try and push past it and push through it. I've got to listen to my body and to pull back and listen to it
      8. Participant 5: it's maybe a warning sign. I think, well, it's a good thing, maybe, the body needs a bit of rest.
      9. Participant 4: I didn't know what I'd really done to it. So, there's been a few different types, really, and I don't know. I never know what I think I've done with it
      10. Participant 6: To me, it means that the area's irritated.
      11. Participant 7 - If I'm over training or something I don't really know, perhaps the rubbing together of the tendons causing mini fractures?
      12. Participant 2 - I don't actually know what's going on. When I feel the pain, I mean I feel it in the base of my achilles but I don't know what's going on. It's kind of ... It just feels like it's too tight I guess. But I got no idea what's actually going on. I mean the physio explained it to me once upon a time but I can't remember what he said.
      13. Participant 2: “It's probably telling me that I'm doing too much. It's probably a warning sign that I need to maybe adjust what I'm doing in some way, shape or form.”
      14. Participant 13: Maybe it's a degenerative thing that has made it worse
      15. Participant 13: On the heel, I feel like it's a pressure point that's pushing on the bone, that's one of the things
      16. Participant 13: the tightening, and pushing the pressure on the bone
      17. Participant 11: The pain indicates to me that perhaps it's weak.
      18. Participant 9: I feel like I'm doing something bad to my body and in the future, it's not sustainable
      19. Participant 4: I feel like I should know more, but I don't.
   3. **Fear Avoidance**
      1. 12/15 participants reduced or stopped physical activity due to fear
      2. 7/15 participants demonstrate fear of tendon rupture
      3. Participant 9 - It's a very frustrating pain, because you can feel it kind of in many things that you're doing, so you're constantly reminded of it, and I'm kind of thinking, "Okay, I can't train at the level I want to train, I won't be able to reach the potential that I feel like I want to and was like to and think I can reach, because I won't be able to train at that capacity or race at that level," so it's more the thought of ... And that's what I love to do, so it's like, "Oh, well maybe I shouldn't do that workout today or tomorrow, because it's gonna hurt and it's not worth it to then be ... I'd rather run when it's healthy ."
      4. Participant 3 - I often just pull out earlier than ... I never let it get that bad, if you know what I mean? I don't really go in as hard. I've got that kind of doubt niggling in the back of my mind about it. That I need to protect it, rather than let it get too bad. So I'm not someone who would take it that far to the edge. I think that's probably more of it, is it just hinders me from going further or harder, or any of those things really.
      5. Participant 11 - And like, so I think that life, because I picked up bike riding and swimming, that’s ok but if for example, if someone told me, "Let's go hiking somewhere.", you know, I wouldn't probably go. I would say, "Nah, I'm busy or maybe I will tell them the truth or some other excuse.
      6. Participant 10 - That's always at the back of my mind I guess, which is why I've pulled back the training and not running a bit because that's ... I don't want it to get to that point. Obviously that's a longer recovery period. Yeah, just around people that have had them, or know someone that has had one. You know, obviously it requires surgery, and a lot of therapy and time off doing your normal routine, day to day activities as well
      7. Participant 5 - I guess I would be careful and mindful of where I'm going, who I'm with, like my crowd and where I'm going to be. Something that could be ... not dangerous, but I'm concerned I'm going to put myself in that situation.
      8. Participant 4 - So, I do a little bit, but I don't try to do too many hills because I'll be pretty sore afterwards. So, I'm steering clear of that a little bit while I'm training for Osaka marathon because I don't want to cause myself too much trouble
      9. Participant 14 - I'm quite certain that if I played table tennis, it would definitely be worse. I'm not even attempting that, because I'm just scared that I might rupture a tendon
      10. Participant 4 - Oh, I did start to play netball again, just casual net ball, earlier in the year, and that just terrifies me about maybe jumping up for a ball or something and it just going. It is definitely a fear. Yeah.
   4. **Treatment Beliefs**
      1. 9/15 participants belief rest is a primary treatment
      2. 9/15 participants belief that strength training is a primary treatment
      3. Participant 1 - I think the heel raises are definitely strengthening my calf muscles and it allows … And explained to me that my calf muscles are not as strong even though they look quite strong. But physically, they’re not as strong. The idea was to strengthen the calf muscles because running requires a lot of calf muscles so that the load even … the load is taken up more by the calf muscles than by the Achilles heel, so they’re different.
      4. Participant 14: And strengthen the muscles surrounding it, and eventually, if I persist with exercises and continue to do them, even if I get better, I should be able to walk really normally
      5. Participant 3 - Yeah, it just feels worn. Like it's just had enough of whatever you're doing to it. Yeah, that's kind of how it feels. It is just saying, rest, rather than do it again. Yeah, I'm not too sure.
2. **Experience with Management Process**
3. **Motivations to seek treatment**
   1. 11/15 participants were motivated to seek treatment by pain and fear of disability/worsening condition
   2. 4/15 participants reported lack of motivation to seek treatment
   3. Participant 2: “When I was motivated, I did. When I was properly into it, I did them very regularly. I was religious with them. I had an alarm three times/day and I was in the gym everyday or every second day and it drove me nuts but yeah, I was motivated because I had these races coming up. But I don't have any races so I'm not motivated to do that and it's very very hard to drag myself away from what I'm doing at work to try and do some of these exercises.”
   4. Participant 14: But, I've joined a gym and I never was a fan was of that kind of thing. So, I'll go. That motivates me. And it's (the gym) around the corner from where I live, so that's really a powerful motivator
   5. Participant 9: I usually just run through little things and they go away, so that's what initially happened, and then I realized, "All right, this isn't going away on its own. It doesn't really seem like a little thing. I guess I'll go get help
   6. Participant 8 - Well, the tipping point is probably when I'm just not able to do the distance and I guess the pain. I start developing pain that doesn't settle after a week or two. Reduced activity, it's still there or it reduces. I would start up slow again and it's still there after probably ... I guess I can't really think about how long I let it rest. Yeah, I probably leave it go for a week or two and then try just lower volume again and if it's still there, that's probably when I'd probably try just basic first aid... I guess I stayed at home first, non-steroidals and I wrapped ice and all that sort of stuff and then rest. Yeah, that's probably my trigger for seeking follow up.
   7. Participant 3 - I don't know what it is. I guess you just get so annoyed with it, or annoyed with yourself at not doing anything about it. That then you just end up doing it. I think I've got better at asking for help now than before. I would've tried to treat stuff myself.
   8. Participant 7 - I think because eventually if the pain increases and stops me doing activities that I really want to, like longer distance running, I'd seek longer term treatment and follow through with it properly.
   9. Participant 7: I think at the point where I feel I can't run through it any more, that it moves from a niggling pain to a, "Oh, this is painful, I actually need to stop and walk.”
   10. Participant 4 - It wouldn't go away and I cracked it one day and I was like, "I need to do something about it." Because I couldn't run very far. Then I went into a physio and then he said I have one of the worst Achilles tendinopathies he'd ever seen, actually.
   11. Participant 10 - And there's still things I want to do in the future, like with running and more marathons, and even doing some ultras and stuff like that. So I don't have a choice but to keep it strong and keep doing those exercises.
4. **Lack/Barriers with motivation**
5. Participant 2: I was motivated because I had these races coming up. But I don't have any races so I'm not motivated to do that and it's very very hard to drag myself away from what I'm doing at work to try and do some of these exercises.”
6. Participant 2 - It's maybe because I continue to ignore all my strength exercises or don’t perform them as often I should and as consistently as I should. Because I'm lazy and unmotivated.
7. Participant 11: some routine is required. You have to incorporate some routine, and sometimes it's hard to start.
8. Participant 15: I failed to go to the gym this morning because I was feeling fed up with myself and so I'm not really in control of any of these things.
9. Participant 4: it's just not enjoyable and seems to be tedious, some of the exercises, sometimes. You just don't want to do it, to tell you the truth.
10. It's not as enjoyable, I think, is probably the biggest barrier.
11. Participant 8 - I think it's the frustration of you want something to happen. You want it to happen now. You're doing all this stuff and it's just very slow progress. I guess it's the frustration of the speed of progress is ... yeah. I guess it's just ... call it motivation to continue.
12. Participant 7 - No, beside from the fact that I was doing it in public and it sometimes looked a bit weird, aside from that it was easy to perform. Mainly home based and not really anything more than those exercises really around the achilles thing anyway. Yeah, I'm not as much of a gym person? I'd try to alternatively find a good strength routine that I could do at home, potentially with some home weights and stuff, I wouldn't be too keen to go to the gym.
13. Participant 14 - But, I've joined a gym and I never was a fan was of that kind of thing, because it's not motivating, whereas if you play a sport, like tennis or table tennis ... You know, it's highly motivating, in many ways, these games.
14. **Experience with passive treatment**
15. 14/15 participants were prescribed or sought passive modalities for management (12/15 for massage, 7/15 for dry needling/acupuncture)
16. Participant 9 - So acupuncture tends to ... I respond really well to that and pretty quickly as well. Obviously massage, anything to loosen up my calf, really. So massage work or acupuncture on my calves. Anything else to make it feel better? I've kind of played around, I don't have ... Those are the two biggest things, but little things that I've kind of depending ... I can't say that they've always helped, but I felt sometimes that they did help was a night splint and icing right after a tough workout or race
17. Participant 4: I've had a lot of massage over the years. I've had dry needling in my calves because my calves and hamstrings are tight and this may be contributing to my symptoms.
18. Participant 13: When the pain along the tendon up to calf was at its worse, the deep tissue massage, I found, was very beneficial.
19. Participant 10- Yes and no. The dry needling definitely, it really did initially help. At that point it wasn't acute tendonitis or anything, that happened after the dry needling. So that kind of helped but because I felt better again I went and did more running, which made it to that next level. Certainly when I first got up in the morning, even after having that on all night, it was still sore to put down flat on the floor so from that perspective that didn't change. It maybe took away some of the swelling or whatever because that's better at the moment.
20. Participant 8 - I guess just my opinion is that the muscle component of it felt tight and that, in my mind, loosened that off and I feel it made the muscle more effective. The shock wave, I imagine it had impact on the way the pain is, has some role in the way the pain is generated and whether or not it has some other impact on contributing to healing or blood flow or whatever. I'm not sure.
21. Participant 14: physiotherapist I usually go to, just manipulating the muscles in my calf.
22. Participant 13: Yeah, they did the deep tissue massage type of thing, and also with the interferential and strapping
23. Participant 8: I've probably had some work on my calf and releasing tension and problems there.
24. Participant 8: I've had some shock therapy in terms of treatment
25. Participant 8: I guess just my opinion is that the muscle component of it felt tight and that, in my mind, loosened that off and I feel it made the muscle more effective
26. Participant 4: Earlier this year when I was sore, I had a bit of ultrasound to try and break up all the tissue and all that sort of stuff
27. Participant 4: I've had a lot of massage over the years. I've had dry needling in my calves because my calves and hamstrings are tight and this may be contributing to my symptoms.
28. **Experience with active treatments**
    1. 6/15 participants believed strength training was the most effective treatment
    2. 13/15 participants were prescribed some form of strength training by HCP’s
    3. Participant 2: “I would say the isometric type exercises and the strength work is been ... When I did it, it was bang on and it solved my problem. Yeah, it was just about ... It was a long road. It was just about consistently doing it, regularly doing it and committing to the treatment.”
    4. Participant 6: I mean, I would say the exercises, just because I think there's ... I think one of the problems of runners is they just think, they don't like think of all the other muscles that are affected in the foot and in the legs.
    5. Participant 2: “full lower body strength exercises and the isometric type exercises with my foot and strength with my leg like gym stuff with my legs.”
    6. Participant 10: my physio has given me just the heel lifts. So store, jump up and release.
    7. Participant 15: I can't be bothered to do the exercises that I've been given, so may actually be my fault.
    8. Participant 14: I'm not sure that the exercise alone, without the shock wave, would've been effective
    9. Participant 1: The only way I could say it is that force yourself to take a rest even when you’re doing well.
    10. Participant 9: I need to take time off and take care of it and come back and have it feel good.
    11. Participant 6 - I mean, I just keep thinking, am I going to have to eventually stop running just to kind of eliminate the pain in the achilles and to not put so much force on it.
29. **Frustration with Providers/Education**
    1. 5/15 participants reported frustration and/or dissatisfaction with medical providers and education.
    2. Participant 13: Online, because it's there, sometimes when you talk to your doctor, or the specialist, it's very limited time, and they don't have the time to explain it properly, and they speak In technical terms. I thought the physio spent a bit more time with you to talk to you about it.
    3. Participant 9: I guess just in the last couple years when it got bad again, then I went to go see a doctor again and didn't really care for what he had to say,
    4. Participant 4: everyone has their sort of different opinions, so it's not, I don't know, not always consistent information at the same time, as well.
    5. Participant 4: You read so much stuff, so it confuses you at the same time.
    6. Participant 12: Confusing for me. Terminologies and things like that. I think even if I read what's in front of me, at the end of it I'm not quite gonna understand it, so I might as well just close the book.
    7. Participant 13 - And every time I've gone to the doctor to complain about it, he's been saying, oh, massage it, or use heel lifts and things like that, and I don't feel that has worked on it, if you know what I mean, and I always feel like, oh, if I was a footballer it'd be fixed in eight weeks' time, if you know what I mean?
    8. Participant 12 - He told me it was stretching and possibly help ease it. As I said, I did go back, but I went back for my back, not for my Achilles. Honestly, I didn't see that much difference. I thought, "I could be doing this for another eight visits," and still be in the same spot, so I pulled the pin, because if I'm gonna feel anything [crosstalk 00:21:01]. I think I did it twice, and the first time I went, didn't do anything. Second time, I didn't feel any different at all, so I thought no. He can stick with my back.
30. **Impact of AT**
    1. **Impact on daily routine**
       1. 15/15 participants reported that their daily routines and activities are affected.
       2. Participant 4 - Because I'm on my feet all day at work, it's painful at work sometimes, but it doesn't really hinder me too much at work, but I suppose when it's really flared-up, there's been things that I obviously don't want to go do. Like a hike or something, I'm like, "Oh, I'd better not because it's sore and you're walking up and down hills all day. It's quite painful
       3. Participant 13 - So, yeah, I think it restricts me in a lot of things that I would be able to do. I don't think I can go out and kick the footie with my son, or ... You know, I manage to ... in pain, to go for a walk with the dog in the evening, if you know what I mean? But-
       4. Participant 5- When I'm walking in the ground, like sand or anything like that, or any uneven surfaces, because I don't want to aggravate it. I don't want to make it worse than what it is, because I just find that when it is really painful, it stings like anything. And that stinging is so painful, it's just like horrible sometimes.
    2. **Impact on running**
       1. 12/15 participants have reduced or stopped running due to AT
       2. Participant 13 - Yeah, as I said, I can't run, walking, initially, I can't walk the great distances that I used to be able to. So, for example, when we go for a walk to get our coffee in the morning, you know, I'll be limping steps behind my colleagues until it loosens and warms up, and then I can walk with them at the same pace. So, yeah, I think it restricts me in a lot of things that I would be able to do. I don't think I can go out and kick the footie with my son, or ... You know, I manage to ... in pain, to go for a walk with the dog in the evening, if you know what I mean?
       3. Participant 2: “I’ve reduced my running. So running too much makes it worse.”
31. **Psychological Impacts**
    1. **Frustrations**
       1. 11/15 participants described frustration and/or annoyance with their condition and its limitations.
       2. Participant 9 - It's a very frustrating pain, because you can feel it kind of in many things that you're doing, so you're constantly reminded of it, and I'm kind of thinking, "Okay, I can't train at the level I want to train, I won't be able to reach the potential that I feel like I want to and was like to and think I can reach, because I won't be able to train at that capacity or race at that level," so it's more the thought of ... And that's what I love to do, so it's like, "Oh, well maybe I shouldn't do that workout today or tomorrow, because it's gonna hurt and it's not worth it to then be ... I'd rather run when it's healthy."
       3. Participant 8 - Well, I think it's just like there's things that I enjoy doing and if I can't do them, now I get a bit frustrated and it's part of what makes me happy and makes me satisfied with things. Yeah, I think it's part of those basic sort of ... You know, you do a nice, long run and you feel quite good after it. I'm not having that experience. I think that satisfaction, the challenge, and all that sort of stuff, I'm just not being able to do and expose myself to and I kind of struggle to find that in other modes when I'm not running.
       4. Participant 15 - Well, it's in my character to ignore things like this. I just accept them as part of getting old. So, it annoys me. It doesn't annoy me as much as other things that's going wrong, but it is slightly embarrassing. I mean, if you have to stand up and you find yourself hobbling, and you think "Oh God, I'm getting old." and it's aligned. But as I said to you earlier, I can't be bothered to do the exercises that I've been given, so may actually be my fault
    2. **Self-blame**
       1. 10/15 participants blame themselves for the cause of their AT
       2. Participant 2 - I'm the only one to blame for it being the way it is. Yeah, I mean it's certainly in my control. I can't blame anyone else for it being the way it is and I've ... It's my decision to do or not do my exercises and things like that. I wish there was a magic pill that I could take to resolve it.
       3. Participant 11 - Yeah, because maybe because I did not, almost nothing about it. Maybe I should have gone to the doctor but my GP was probably going to offer cortisone, which I was kind of scared about. And, but it was basically mainly my negligence, and also since I have it now, I really should start some exercise regimen, you know, specifically what the physio gave me last time. I should really do it more often, not only when I remember once a week or something like this.
       4. Participant 11: I failed to really maintain it properly. I should have done more of those exercises prescribed
       5. Participant 2: I'm the only one to blame for it being the way it is.
       6. Participant 4: So, I've probably got myself to blame for it.
    3. **Annoyed**
       1. Participant 3 - I don't know what it is. I guess you just get so annoyed with it, or annoyed with yourself at not doing anything about it. That then you just end up doing it. I think I've got better at asking for help now than before. I would've tried to treat stuff myself.
       2. Participant 15 - Well, it's in my character to ignore things like this. I just accept them as part of getting old. So, it annoys me. It doesn't annoy me as much as other things that's going wrong, but it is slightly embarrassing. I mean, if you have to stand up and you find yourself hobbling, and you think "Oh God, I'm getting old." and it's aligned. But as I said to you earlier, I can't be bothered to do the exercises that I've been given, so may actually be my fault
       3. Participant 15 - No. It's just occasionally if I'm sitting at my desk and I notice it and I'll just rub it while I'm carrying on, but I don't think it prevents me from doing anything. And it tends to be when I'm sitting down and in that it's annoying. I just sit there, fiddling with it.
32. **Future / Prognosis**
    1. **Hopes of future**
       1. 10/15 participants have a positive outlook on their future prognosis
       2. Participant 14: It can be relieved, but there's nothing you can do about flat feet. I'm born like that. If I can keep up the exercise, I feel it's better. But I also recognize that I'll probably have to do these exercises for the rest of my life. That's how life is. I'm not gonna play tennis again. I'm not sure that I'll be able to play table tennis
       3. Participant 10 - Because it's not ruptured, and it's not overly bad. Like I can walk fine now, and it has improved, so there has been improvement. I guess with the treatment and no running. So I think it'll just be a matter of time when I build up those exercises that it will come good.
       4. Participant 8 - I see myself better. I think I feel like I'm very much in the last stages of this problem. I see myself better and I think of one of those things I progress with learning ways to ongoingly prevent these sort of injury. Yeah, I still see myself running.
       5. Participant 10: So I think it'll just be a matter of time when I build up those exercises that it will come good.
       6. Participant 9: I hope that in the next couple months, I can do the right stuff for it to feel healthy and strong and that can carry through for training for the next couple years.
    2. **Negative beliefs regarding prognosis**
       1. Participant 14: It can be relieved, but there's nothing you can do about flat feet. I'm born like that
       2. Participant 14: That's how life is. I'm not gonna play tennis again. I'm not sure that I'll be able to play table tennis
       3. Participant 5: I don't know about being cured.
       4. Participant 4: I don't think I'll be better or worse. I feel like I'm gonna continue being the same.
       5. Participant 12: I personally think it's not gonna go away
    3. **Positive self-efficacy**
       1. Participant 3 - Yeah, definitely. Yeah, definitely. It used to be worse, because I literally only ran. That was the one thing, whereas now I try to diversify a bit. I'm not any good at them, but I just, I do swim a bit sometimes. I just commute cycle, but at least I'm slightly active. So last time, when I got injured and I couldn't really run that much, that's when I started swimming again. I tried to put a positive spin on it. Try and be like, "Oh, do something different."
       2. Participant 10 - I guess because I've just maybe come to the conclusion that it's not going to get better unless I treat it, and take professional advice of my physio and continue with what she's saying to do and also not to do.
       3. Participant 5 - I guess it's just a case of just like doing all the exercises and just kind of managing it, because any time when I don't keep up doing my exercises, then it starts weakening. And the amount of running that I do, especially when I'm marathon training as well. It's something that's not going to go away, and if I don't keep up doing the exercises, it's just going to weaken. I'm always mindful to keep it strong and keep it going well, I guess
       4. Participant 5 - And my physio is really great, she helps out. If I have any concerns, she just drops me a line, and make an appointment to see her. And we try and figure out, try and sort it out what I've been doing recently, or maybe not enough gym work, and not enough of the exercises and stuff like that, so then it's kind of weak. And then I'm like, okay, I've just got to re-concentrate and put it back into my mind again so that I am able to make it get better and heal and get better so I can keep running.
       5. Participant 14 - If I can keep up the exercise, I feel it's better

**References**

1. Braun V, Clarke V. Thematic analysis. In: Cooper H, Camic PM, Long DL, Panter AT, Rindskopf D, Sher KJ, editors. APA handbook of research methods in psychology, Vol. 2. Research designs: Quantitative, qualitative, neuropsychological, and biological. Washington, DC: American Psychological Association; 2012. pp. 57–71.
2. Mc Auliffe S, Synott A, Casey H, Mc Creesh K, Purtill H, O'Sullivan K. Beyond the tendon: Experiences and perceptions of people with persistent Achilles tendinopathy. Musculoskeletal Science and Practice. 2017;29: 108-114.
3. Thorne S, Kirkham SR, MacDonald‐Emes J. Interpretive description: a noncategorical qualitative alternative for developing nursing knowledge. Research in Nursing & Health. 1997;20(2): 169-177.
